# Physician workforce planning in Ontario must move from short-term reactivity to long-term proactivity

**Published:** 2018-05-31

**Authors:** Brandon Tang, Linghong Linda Zhou, Keyvan Koushan

**Affiliations:** 1 Faculty of Medicine, University of Toronto, Ontario, Canada; 2 Dalla Lana School of Public Health, University of Toronto, Ontario, Canada; 3 Department of Dermatology and Skin Science, University of British Columbia, British Columbia, Canada; 4 Department of Ophthalmology and Vision Sciences, University of Toronto, Ontario, Canada

This year, there were 115 Canadian Medical Graduates (CMGs) who went unmatched to a residency position after the second and final iteration of “the match,” a process overseen by the Canadian Resident Matching Service (CaRMS).^1^ This latest count, another increase, is part of a series that has been rising dramatically: from 11 in 2009, to 46 in 2016, 68 in 2017 and now 115 in 2018.^2^

Unmatched CMGs are unable to complete a necessary extension of their education, meaning that a substantial amount of time, energy, and taxpayer dollars are being invested into training physicians who may eventually be unable to serve their communities as independent practicing physicians. This large number of unmatched CMGs raises questions about the adequacy of physician workforce planning. Although our analysis focuses on the province of Ontario, as all Canadian provinces redefine their strategy for physician workforce planning,^3^ medical school and residency planning should be key considerations, rather than afterthoughts, in the integral process of aligning physician training to population needs.

## A historical look at workforce planning

Determining the ideal number of both undergraduate and postgraduate medical trainees in Ontario has historically been a struggle. Over the last few decades, aggressive changes to medical school enrollment in response to physician supply projections have created alternating physician surplus and undersupply. In 1991, the Barer-Stoddart Report recommended a 10% reduction in medical school positions after confirming the growing consensus of a physician oversupply.^4^ In 1993, the Ontario government responded with a 12% reduction in enrollment, motivated in part by the potential to reduce healthcare costs.^5^ This was consistent with the broader political atmosphere of austerity, as evidenced by the recently imposed ban on physicians from extra-billing beyond the provincial fee schedule.^6^ By 1999, however, concerns of physician undersupply arose, underscored by the McKendry Report,^7^ which prompted a similarly aggressive reaction in the opposite direction. From 2000 to 2017, annual Ontario medical school admission numbers have nearly doubled from 571 to 955.^8^

Short-sighted changes were similarly seen in postgraduate education. In 2015, the Ontario government announced a plan to eliminate 50 residency positions over the next two years, in an effort “to manage the risk of an oversupply of physicians in the future.”^9^ Consultation with key stakeholders appeared to be limited, given the criticism mounted by the Ontario Medical Association and multiple medical student advocacy groups,^10^ while no concomitant decrease in medical school admissions was planned. In stark contrast, the Ontario government announced in April 2018 that they planned to “fund additional residency positions for medical school graduates who have completed their undergraduate training at an Ontario medical school […] to ensure that all unmatched [Ontario] graduates are placed”.^11,12^ This abrupt policy reversal will cost Ontario up to $23 million over 6 years.^11^

Proceeding further along the spectrum, similar troubled histories have been documented in the physician job market.^13^ Many trainees are “experienc[ing] significant difficulties obtaining a [job] position [in a certain geographical location]”.^14^ Along the same lines, a survey administered to Quebec residents in 2014 found that 77.9% of respondents “believed [that] there are not enough job opportunities for the number of trainees.”^14^

Altogether, this demonstrates that it is important to understand and question the practices that have governed physician workplace planning in Ontario. These historic trends suggest that determining the number of both undergraduate medical school entrants and postgraduate residency positions have largely been based on myopic practices to cope with existing surpluses and deficits in physician supply, rather than taking a long-term view. In addition to creating a bottleneck for graduating medical students to enter residency, these reactive practices are likely contributing to the emerging problem of physician unemployment, a reality faced by 16% of Canadian graduates from specialist residency programs in 2013.^15^ However, it is ultimately patients who suffer most: while competent medical trainees and physicians wait in the wings to enter practice, wait times continue to lengthen.^16^

## More than just a number’s game: A second mismatch emerges in physician workforce planning

There is a second emerging imbalance stemming from medical trainee and physician preferences for a particular specialty and/or geographic location. This issue is complex, controversial, and much less discussed, though it may actually be contributing to a larger portion of the problem. To highlight this point, there were 198 CMGs who went unmatched after the first iteration, according to data published by CaRMS this year.^1^ At the same time, there were 228 unfilled positions across the nation which would be rolled over and ideally filled in the second iteration.^1^ Despite this, 46 of the 198 unmatched CMGs chose not to participate in the second iteration.^1^ Of those who did participate, a large majority of the 115 still failed to match^1^ for reasons which are not entirely clear. In contrast, the top five specialties in highest demand had an average supply-to-demand ratio of 0.53 by CMGs in the same year,^1^ reflecting the competitiveness of certain specialties or, in other words, the mismatch between trainees’ preferences and positions available in these specialties.

Perhaps, as highlighted by Andre Picard in a recent article,^17^ we need to earnestly examine why specialties with the greatest need for service (e.g., primary care specialties) are less preferred by CMGs. Mr. Picard suggests that it may be because “we’re not attracting the right people to medical school,” or in other words, a factor of *who* we select. However, having recently emerged from the training system ourselves and backed by literature that proposes contributing factors,^18^ it would be unwise to disregard the role that the medical system itself plays in shaping values, goals, and career aspirations, thereby contributing to this situation.

As described earlier, the Ontario government aims to create additional residency positions this upcoming year for all unmatched Ontario medical school graduates.^11^ The interest and uptake of these positions, as well as the profile of the students who fill them (e.g., year and number of times unmatched statistics, initial first choice specialty preference, first choice geographical location), will be a natural experiment to understand how the forces of position availability and student specialty preferences interact.

Adding to the complexity behind unmatched medical graduates is the fact that there are a large number of positions in Quebec, which require specific fluency in French. Given that a significant proportion of French-speaking students choose to match out of province to English-speaking positions,^1^ this effectively means that there are not enough available positions for English-speaking graduates. Underscoring this point, there were 109 unfilled residency spots in Quebec out of a total 228 (47.8%) after the first iteration^19^ and 69 out of 78 (88.5%) after the second iteration,^20^ demonstrating an inability to fill these positions.

Thus far, we have highlighted mismatches between the volumes of positions at different stages of medical training, as well as between trainee preferences and position availability. That said, in recent years, there has been some success in closing the gap. The Northern Ontario School of Medicine (NOSM) is a prime example of harmonized planning between undergraduate medical school admissions, postgraduate residency positions, physician workforce allocation, and population needs. By recognizing that physicians from rural backgrounds were more likely to practice in rural areas,^21^ the McKendry Report recommended the establishment of NOSM in 2001 to help address the growing underserved rural regions in Ontario.^7^ Early reports suggest that NOSM is succeeding in accomplishing its goals, with 61% of NOSM-graduated family physicians practicing in northern Ontario in 2014.^22^ Moreover, the greater the number of years a trainee spent at NOSM in their undergraduate and postgraduate training, the greater the odds they would ultimately practice in rural northern Ontario,^22^ indeed reflecting a success on both fronts.

## Planning for the future: The importance of coordination along the continuum of medical education

The rising number of unmatched CMGs may be a symptom of a larger problem; that is, poor coordination between UGME, PGME, governmental strategy, and population needs. In turn, solutions should treat the underlying cause, as opposed to the symptoms that appear most urgent.

We need an integrated and holistic approach. A recent review of over sixty years of published studies highlighted the need for undergraduate medical education (UGME), postgraduate medical education (PGME), and provincial governmental bodies to work together to streamline physician workforce planning to better serve population needs.^23^ In line with this philosophy, a pan-Canadian Physician Resource Planning Task Force, co-chaired by the Ministry of Health and the Association of Faculties of Medicine of Canada (AFMC), was established in 2013 to develop a national approach to physician workforce planning.^24^ More recently, the Ontario government included “align education” as one of its key workforce planning approaches in May 2016,^3^ while the AFMC published recommendations to reduce the number of unmatched CMGs in January 2018.^25^

Ultimately, any strategy to align the planning of medical training to population needs will involve processes that will take years for results to emerge. A long-term and unified strategy is required that considers the entire continuum of training – from undergraduate medical school enrollment to postgraduate positions to physician employment – all within the broader context of the healthcare needs of our communities.
